# Improvement of Young and Elderly Patient’s Knowledge of Heart Failure After an Educational Session

**DOI:** 10.4137/cmc.s2357

**Published:** 2009-04-20

**Authors:** Jérôme Roncalli, Laurence Perez, Atul Pathak, Laure Spinazze, Sandrine Mazon, Olivier Lairez, Daniel Curnier, Joëlle Fourcade, Meyer Elbaz, Didier Carrié, Jacques Puel, Jean-Marie Fauvel, Michel Galinier

**Affiliations:** 1 Rangueil University Hospital, Department of Cardiology and Heart Failure Educational Centre (Centre d’Education pour Patient Insuffisant Cardiaque (CEPIC)), Toulouse, France.; 2 INSERM U858-I2MR, Hôpital de Rangueil, Toulouse, France.; 3 Service de Pharmacologie Clinique, Faculté de Médecine Toulouse, France.; 4 Deceased.

**Keywords:** heart failure, educational session, knowledge in heart failure, elderly patients

## Abstract

**Background:**

Interest in the role of patient education sessions for optimizing the management of heart failure (HF) is increasing. We determined whether improvements in young and elderly patients’ knowledge of HF and self-care behavior could be analyzed by administering a knowledge test before and after an educational session.

**Methods:**

Stable heart failure patients (n = 115) were enrolled in a prospective cohort study from our Heart Failure educational centre in a university hospital. Patient knowledge of six major HF-related topics was assessed via a questionnaire distributed once before an educational session and twice afterward. Each answer was assigned a numerical value and the final score for each topic could range from 0 to 20. Scores ≥ 15/20 were considered representative of a good level of knowledge.

**Results:**

The level of knowledge was low (9.7/20) before the educational session but was significantly higher (16.3/20) during the 1st quarter after the session, and this benefit was maintained for up to 12 months (16.6/20). Knowledge levels increased in both younger and elderly patients, and the number of patients who had a good level of knowledge also increased after the educational session.

**Conclusion:**

This study confirms that an HF knowledge test is feasible and that educational sessions improve the knowledge and self-management of both younger and elderly patients.

## Background

Heart failure (HF) is a major, growing public health problem. The quality of life among patients with HF tends to be poor^1^ and comparable to that of patients with many forms of cancer.^2^ Although a range of therapeutic strategies (e.g. ACE inhibitors,^3^ beta-blockers)^4^ have improved patient survival, it is often difficult to apply these strategies in elderly and fragile patients.^5^ Even with appropriate treatment, disease progression leads to recurrent hospitalizations, which contribute significantly to the economic burden associated with HF.^6^,^7^ Hospital activity is the greatest component of HF-related costs,^8^–^10^ and although hospitalizations are sometimes crucial for management of congestive HF, a large proportion of them can be avoided.^11^ As the population of patients with HF ages and their frequency of readmission increases, there is an increasing economic need to limit hospital activity.^12^

Patient education is generally recognized as an important component of comprehensive management programs for chronic conditions, and there has been an increasing interest in the role of patient education sessions for optimizing the management of HF. Therapeutic interventions combined with an educational session have been linked to improvement in patients’ self-care behavior.^13^ Conversely, lack of knowledge leads to low compliance and is a major contributor to poor quality of life and hospital readmissions. At present, it is difficult to assess the benefits of educational sessions for HF. The establishment of a “knowledge test” could provide insight into patients’ needs and help practitioners prescribe educational sessions. We have prospectively studied the effects of an education session on a cohort of HF patients. The goal of this study was to evaluate the validity of a test designed to assess the knowledge of young and elderly HF patients and to identify the potential benefit of educational sessions for improving patients’ HF condition.

## Methods

### Patients and setting

Patients from the Heart Failure Educational Centre of our Department of Cardiology at the University Hospital of Rangueil in Toulouse (France) who had stable HF were solicited to enroll in this study. All patients gave informed consent, our institutional review committee approved the protocol, and our study was conducted according to principles of the Declaration of Helsinki. Nurses, research associates, and medical doctors were trained to ensure that only patients with systolic left ventricular dysfunction were enrolled. Patients who were unable to speak French and patients with transient HF, severe psychiatric illness, or cognitive impairment were excluded. All baseline data was obtained immediately after enrollment. Functional capacity was systematically evaluated by using the New York Heart Association (NYHA) classification,^14^ the 6-minute walk test, and (when possible) peak oxygen consumption to assess the severity of HF.

### Intervention

Early in 2001, a multidisciplinary team implemented an educational session designed to promote the self-management of patients with HF. Hospitalized patients with HF were educated by staff nurses through the use of brochures available from the French Cardiology Society and tools developed by physicians with expertise in the field of HF. Patients who did not participate in the educational program received assistance from a social worker or dietician when problems occurred.

Patients who were enrolled in the Heart Failure Educational Centre attended a multidisciplinary educational session for one day at the end of their hospitalization period either before discharge or independent of their hospitalization. This multidisciplinary educational session was established in collaboration with physicians, nurses, dieticians, physiotherapists, and medical social workers, all of whom were specialists in both therapeutic education and HF.

### Measurements

The knowledge test was a self-administered questionnaire prepared in collaboration with the nurses who led the educational sessions. The test enabled us to assess patients’ knowledge of HF and their self-care behavior. Patient knowledge was analyzed for each of six major topics: HF condition, clinical evaluation (signs and symptoms of worsening HF), physical activity, medical follow-up, diet, and medical treatment; the Minnesota Quality of life Score was also assessed. Questions were presented in multiple-choice format, and each answer was assigned a numerical value that yielded a final score ranging from 0 to 20 for each topic. We considered a final score ≥ 15 to indicate a good level of knowledge. The test is available at the address of the corresponding author.

The knowledge test was handed out before the educational session (T0), then the same test was redistributed to patients (with a prepaid return envelope) during the first quarter after the educational session to analyze the short-term outcome (T1), and again during the fourth quarter to analyze long-term outcome (T2).

### Statistical analysis

Continuous data was expressed as mean ± standard deviation, and discrete or categorical data was expressed as the number and percentage of patients in each category. Comparisons between mean values were evaluated with the Student t-test, and changes in test scores from baseline to follow-up were evaluated via repeated-measure analysis of variance. A *P*-value < 0.05 was considered statistically significant.

## Results

### Clinical characteristics of heart failure patients

Demographic information for the 115 patients with stable systolic HF who attended an educational session is displayed in Table 1. Functional evaluations were performed before the educational session for 78 patients (68%), including patients (60%) who underwent a standardized 6-minute-walk distance test and patients (55%) who underwent a cardiopulmonary exercise test to measure peak VO^2^ exercise oxygen consumption; 30% of patients walked less than 300 meters and 45% patients had a peak VO^2^ less than 14 mL/kg per min.

### Patient’s knowledge level before the educational session

**Figure 1.**
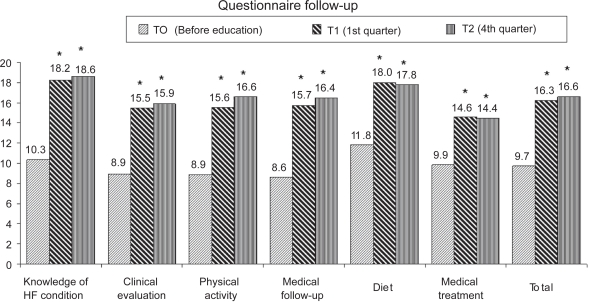
Knowledge levels of patients with heart failure before and after attending an educational session. Patients completed a questionnaire that assessed their knowledge in each of six topics: heart failure condition, clinical evaluation, physical activity, medical follow-up, diet, and medical treatment. Scores could range from 0 (minimum knowledge) to 20 (maximum knowledge). Both short-term (first quarter) and long-term (fourth quarter) outcomes were assessed. **Abbreviation:** HF, heart failure. *T1 vs. T0; T2 vs. T0; *P* < 0.05.

Patient knowledge was low before the educational session (Fig. 1). At baseline, 33 patients (28.7%) had a good knowledge for HF condition, 8 patients (7.0%) for evaluation of clinical signs, 35 patients (30.4%) for physical activity, 14 patients (12.2%) for knowledge of medical follow-up, 36 patients (31.3%) for diet, and 18 patients (15.7%) for medical treatment.

### Follow-up

Short-term (first quarter) outcome was evaluated in 106 patients (92.2%); 3 knowledge tests were missing because of patient deaths, 4 because patients had received transplantations, and 2 were not returned. Long-term (fourth quarter) outcome was evaluated in 70 patients (67.3% of 104 living patients who had not received transplantations); 2 additional patients had died, 2 had received transplantations, and the remainder did not return the test.

**Figure 2.**
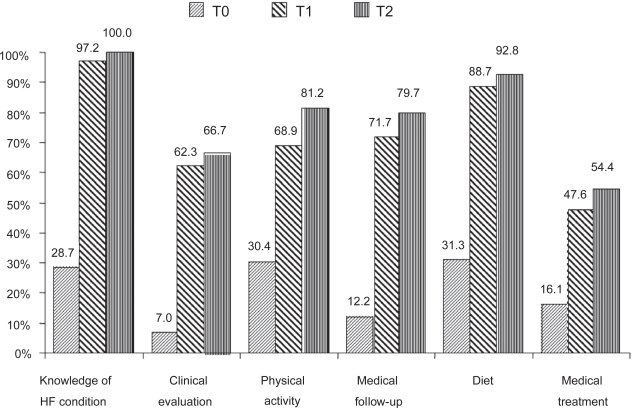
Percentage of patients with a good level of knowledge (score ≥ 15/20) for all topics before (T0) and after the education session (T1, first quarter; T2, fourth quarter). T0 versus T1 *P* < 0.0002; T1 versus T2 *P* = 0.53; T0 versus T2 *P* < 0.0001 for all topics.

The knowledge test enabled us to compare patient knowledge levels before and after the educational session. The level of knowledge had improved in all topics during the first quarter after the educational session (Fig. 1), and this benefit was maintained in the fourth quarter. The benefit of education was also evidenced by an increase in the number of patients who displayed a good level of knowledge (score ≥ 15) for up to 12 months after the educational session (Fig. 2). Knowledge and self-care behavior changes were also evaluated by asking patients specific questions concerning their understanding of HF and changes in clinical evaluations, medical visits, and medical and non-medical therapies (Table 2). Our most striking finding was that patients did not realize HF is a chronic condition until after initiating the educational session. The most difficult topic of education was teaching patients how to modify their drug use as their clinical statuses changed; after the educational session, less than 40% of patients knew which medications are efficient treatments for worsening cases of HF.

**Figure 3.**
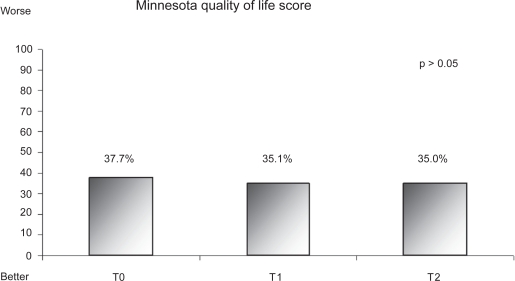
Patient quality of life before (TO) and after (T1, first quarter; T2, fourth quarter) the education session as measured via the Minnesota Quality of Life score. Scores could range from 0 (best) to 100 (worst).

Self-care test scores concerning medical follow-up, diet, and medicine habits were better in younger patients (<65 years) than in elderly patients (≥65 year-old) (Table 3, *P* < 0.05). Nevertheless, the benefit of educational sessions improved from baseline (T0) to the first quarter (T1) for both groups (T0 vs. T1: *P* < 0.05). Every patient displayed greater knowledge after the educational session, and the mean increase from baseline (T0) to the first quarter (T1) did not differ significantly between age groups (*P* = ns). Knowledge of physical activity was not dependent on age (Table 3). Patients were satisfied with the educational session (data not shown), but we found no evidence of global improvement in quality of life (Fig. 3).

## Discussion

The results of this study demonstrate that i) patients with HF have a low level of knowledge of their condition and how to manage it, ii) a knowledge test is feasible for patients with HF and can provide a useful assessment of the need for educational sessions, iii) HF educational sessions significantly improve the knowledge of patients as assessed by this test, and iv) educational sessions benefit both elderly and younger patients.

Before attending the educational session, our patients’ level of knowledge was similar that of patients in a recent report.^19^ Although 2/3 of our patients knew how the heart works before the session, only 10% understood that HF is a chronic condition. These results are consistent with those published by Ni et al. who found that 37% of patients knew little or nothing about congestive HF, 49% knew some, and only 14% knew a lot.^15^ Elderly patients had more difficulty recognizing the link between clinical signs of HF and heart performance, which is consistent with results from another study that found an association between advanced age and lower levels of HF knowledge.^20^ Although patients over 65 may have had difficulty understanding their condition and medical treatment, their improvement in self-care behavior after the educational session was similar to that of younger patients. Thus, educational programs can be beneficial for all patients, regardless of age. In addition, Ni et al.^15^ showed that patients had better knowledge of their condition if they were married, which is why we offer educational sessions to both our patients and their spouses.

The goal of patient education is to encourage positive changes in self-care behavior, which should improve as patients learn more about their disease. For this reason, our knowledge test included questions about aspects of HF ranging from physiology and pathology to drug therapy. Moreover, our knowledge test is reproducible, and this is the first study to reveal an increase in both a knowledge-test score and in the percentage of patients with a good level of knowledge (final score ≥ 15). Thus, this test could be useful for follow-up of patients with HF and for assessing the benefits of educational sessions. One advantage of our test is the breadth of topics evaluated, including HF condition, symptoms, dietary habits, medical follow-up, and physical activity. The European Heart Failure Self-Care Behavior Scale, by comparison, is a 12-item questionnaire that covers only aspects of self-care behavior (symptoms and hygienic-dietetic rules).^16^ Riegel et al.^17^ have proposed a test based on the recognition of six major HF symptoms (asthenia, dyspnea at rest and at stress, weight increase, edema and palpitation) and on a patient’s ability to evaluate and modify treatment. Another simple and fast assessment, introduced by Bennett et al.^18^ evaluates patients according to scales of medication and dietary compliance.

Because improvement of HF symptoms involves many factors, including both medical and non-medical treatments, the benefits of education are difficult to analyze. However, the most frequent cause of decompensated HF is the lack of appropriate compliance to medical treatment and hygienic-dietetic rules (e.g. adaptations in physical activity, salt intake, liquid intake), which is likely to improve as patients learn more about their condition. Our educational sessions encourage adherence to medical recommendations, thereby making treatment more effective; however, knowledge alone does not ensure self-care compliance.^15^

Since the introduction of more “user friendly” tools, the use of health-related quality of life measures for assessments of patients with HF has increased, which reflects the desire to know how patients feel and how they respond to treatment. We observed no significant global improvement in the quality of life as assessed by the Minnesota Quality of Life Score, which is designed specifically for evaluation of patients with HF. This finding conflicts with a report from the Auckland Hospital;^21^ however, the physical dimension of quality of life, assessed in a subscale of the Minnesota Score, showed a significant greater improvement from baseline to 12 months in the intervention group than in the control group. Inversely the psychosocial dimension of quality of life did not improve because of the social pressures associated with a chronic condition or the financial needs caused by physical and dietetic adaptations (data not shown).

### Clinical implications

The prevalence and incidence of HF are growing because of the improved survival of patients treated for myocardial infarction or hypertension, which are the most frequent causes of HF. Hospital admissions for HF have increased consistently over the last two decades, and the total expenditure on HF ranges from 1% to 2% of all healthcare costs.^23^ Any changes in survival that occur secondary to the application of a new treatment strategy have important implications for long-term healthcare costs. Thus, there is an urgent need for strategies to reduce hospital admissions and to provide the best guidance for HF patients.^24^ We believe that educational sessions benefit both young and elderly patients and can lower the costs of HF by reducing the number of hospitalizations.^22^

Current nonpharmacological approaches to HF therapy incorporate interventions by a multidisciplinary team composed of nurses, physicians, social workers, dieticians, physiotherapists, and other specialists. As with other chronic conditions (e.g. diabetes, hypertension, asthma), our educational sessions for HF encourage patients to understand their chronic condition and to improve their self-care behavior, thereby decreasing the rate of hospitalization. This knowledge is crucial because patients are likely to experience better long-term outcomes if they appreciate the chronic nature of HF. Patients will also benefit by understanding the relationship between HF and clinical signs such as edema, which could encourage them to be more attentive to their legs and weight. To facilitate the development of educational sessions for HF performed in either regional networks or local hospital departments, we need to design educational sessions that are dedicated to HF and to develop standardized educational tools for multicenter evaluations^25^,^26^ of both young and elderly patients.

### Limits of the study

The patient population was not large enough to enable analysis of morbidity or mortality; however, this was not a goal of the study. Interpretation of our results is also limited by the relatively young age of our study population (mean age 56); most patients hospitalized for HF are likely to be older. Nevertheless, our results indicate that the self-care behavior of both our younger and elderly patients improved equally. Our findings are also limited because the test was self administered in the patient’s home, so we do not know how long they took to answer the questions or if they considered their answers to the initial test when responding to the follow-up questionnaires. Lastly, although the questionnaire appears well adapted to our HF patients, it may not be appropriate in regions where patients’ life habits, culture, and socioeconomic conditions differ.

In conclusion, a knowledge test for patients with HF is feasible, can be used to assess the effectiveness of educational sessions, and may improve patient self-management. Both elderly and younger patients benefit from educational sessions.

## Figures and Tables

**Table 1.** Demographic characteristics.

**Patients**	**(n = 115)**
Age (y), mean ± SD	56 ± 14
Married, n (%)	84 (73.0)
Male, n (%)	92 (80.0)
Hypertension, n (%)	50 (43.4)
Diabetes, n (%)	30 (26.1)
Hyperlipidemia, n (%)	57 (49.6)
NYHA 1, n (%)	3 (2.6)
NYHA 2, n (%)	59 (51.3)
NYHA 3, n (%)	47 (40.9)
NYHA 4, n (%)	6 (5.2)
Nonischemic, n (%)	60 (52.2)
Body mass index (kg/m^2^), mean ± SD	25.7
6 minute-walk distance (m), mean ± SD	335 ± 97
Peak VO^2^ (mL/kg per min), mean ± SD	17 ± 8
Ejection fraction (%), mean ± SD	28 ± 9

**Abbreviations:** NYHA, New York Heart Association functional class; Peak VO^2^, peak oxygen consumption.

**Table 2.** Patient knowledge.

	**% Patients**		
	**T0**	**T1**	**T2**
*Knowledge of condition*			
Patients who know and understand the heart pump mechanism	72%	96%	99%
Patients who know that heart failure is a chronic condition	10%	80%	81%
Patients who know the etiology of their heart failure	67%	84%	88%
*Clinical evaluation*			
Patients who weigh themselves at least once a week	71%	87%	76%
Patients who know their weight	97%	100%	96%
Patients who look for signs of or increase in oedema at least once a week	53%	87%	82%
Patients who can note their NYHA status	12%	82%	81%
*Medical visit changes*			
Patients who go to the physician in case of increase in dyspnea	64%	96%	97%
Patients who go to the physician in case of increase in weight	30%	88%	92%
Patients who go to the physician in case of increase in oedemas	43%	94%	99%
Patients who go to the cardiologist at least once a quarter	41%	74%	68%
*Medical and non medical therapeutic changes*			
Patients who practice physical activity at least twice a week	57%	83%	91%
Patients who have a restricted sodium diet adherence	68%	98%	99%
Patients who know which pills are efficient in case of worsening heart failure	25%	38%	36%

**Abbreviations:** T0, Before educational session; T1, first quarter after educational session; T2, fourth quarter after educational session. NYHA, New York Heart Association functional class.

**Table 3.** Relationship between level of knowledge and age.

		**≥65 year-old (n = 37)**	**<65 year-old (n = 78)**
Knowledge of HF condition	T0	10.21	10.34
	T1	18.33	18.34
Clinical evaluation	T0	7.14	8.82
	T1	14.11	15.73
Physical activity	T0	9.25	9.24
	T1	15.09	16.13
Medical follow-up	T0	7.16	9.24*
	T1	14.99	16.13*
Diet	T0	10.21	12.56*
	T1	17.24	18.20*
Medical treatment	T0	8.45	10.47*
	T1	13.16	15.10*

**Abbreviations:** T0, before educational session; T1, first quarter after educational session; HF, Heart Failure. T0 versus T1: *P* < 0.05 for all topics in each group. Mean increase from baseline (T0) to first quarter (T1) did not differ significantly between age groups (*P* = ns).

* *P* < 0.05 (≥65 year-old versus < 65 year-old).
